# Dietary Sanguinarine Mitigates *Clostridium perfringens*-Induced Enteric Challenge in Broiler Chickens: Effects on Productive Performance, Metabolic Status, Antioxidant Capacity, Immune Response, and Intestinal Health

**DOI:** 10.3390/life16091464

**Published:** 2026-09-01

**Authors:** Ali R. Al Sulaiman, Ala E. Abudabos

**Affiliations:** 1 Environmental Protection Technologies Institute, Sustainability and Environment Sector, King Abdulaziz City for Science and Technology, P.O. Box 6086, Riyadh 11442, Saudi Arabia; 2 Department of Food and Animal Sciences, College of Agriculture, Tennessee State University, Nashville, TN 37209, USA

**Keywords:** antibiotic alternative, broiler chickens, *Clostridium perfringens*, immune response, intestinal health, sanguinarine phytobiotic

## Abstract

This study evaluated the efficacy of graded dietary sanguinarine as a phytobiotic alternative for mitigating *Clostridium perfringens*-induced enteric challenge in broiler chickens. A total of 360 newly hatched Ross 308 broiler chicks were randomly assigned to six treatments: a non-challenged negative control, a challenged positive control, a challenged group supplemented with avilamycin at 0.10 g/kg, and three challenged groups supplemented with sanguinarine at 0.06, 0.09, or 0.12 g/kg diet. Birds were reared for 35 days, and all challenged groups received repeated oral inoculations with *Clostridium perfringens* following an initial *Eimeria* spp. challenge. Compared with the positive control, all sanguinarine doses improved cumulative average daily gain, feed conversion ratio, and European production efficiency factor to levels comparable with the negative control and avilamycin groups. Sanguinarine at 0.09 and 0.12 g/kg restored dressing percentage, whereas all doses improved breast yield and increased relative bursa weight beyond both control and avilamycin values. All sanguinarine treatments normalized serum total protein, glucose, total antioxidant capacity, and superoxide dismutase activity, with stronger recovery of superoxide dismutase than avilamycin. In the ileum, all sanguinarine doses increased villus length above the avilamycin and negative control groups, while maintaining comparable villus width. Sanguinarine also improved immune status by restoring serum immunoglobulin Y, interleukin-2, and interferon-gamma concentrations, reducing interleukin-1 and interleukin-6 more effectively than avilamycin, and fully restoring immunoglobulin A at the highest dose. Moreover, sanguinarine reduced harmful bacterial populations and promoted beneficial ileal bacteria across the tested inclusion levels. Overall, dietary sanguinarine, especially at 0.09–0.12 g/kg, may serve as a promising non-antibiotic nutritional strategy for improving broiler resilience under enteric challenge conditions.

## 1. Introduction

The global poultry sector is undergoing a major transition driven by the need to maintain high productivity while reducing dependence on antibiotic growth promoters. This shift has been accelerated by concerns over antimicrobial resistance, drug residues, regulatory restrictions, and growing consumer demand for poultry products raised without routine antibiotic use [1]. In modern broiler production, genetic selection has produced birds with rapid growth, high feed intake, and intensive metabolic demands; consequently, intestinal health has become a central determinant of productive efficiency and flock resilience [2]. Because the intestine is the primary site of nutrient assimilation and a major immunological interface, even subtle disturbances in mucosal function can result in measurable losses in growth, feed efficiency, and flock uniformity [3].

Necrotic enteritis remains one of the most economically important bacterial enteric diseases affecting commercial broilers, particularly in antibiotic-reduced or antibiotic-free production systems [4]. The disease is primarily associated with *Clostridium perfringens* (*C. perfringens*), a Gram-positive, anaerobic, spore-forming bacterium that can proliferate rapidly in the intestinal tract when host, dietary, microbial, and environmental conditions are favorable [5]. Clinical outbreaks are characterized by intestinal necrosis and increased mortality, whereas subclinical infections often persist without obvious clinical signs, making them difficult to detect before production losses become evident [6]. In both forms, *C. perfringens*-associated enteric disturbance can reduce absorptive surface area, disrupt intestinal barrier function, stimulate mucosal immune activation, and impair the efficiency with which dietary nutrients are converted into body tissue [7,8].

The withdrawal or restricted use of antibiotic growth promoters has intensified the search for alternative nutritional strategies capable of stabilizing the intestinal environment while preserving growth performance [9]. Among these alternatives, phytobiotic feed additives have gained considerable scientific and commercial interest because they contain diverse bioactive compounds, including alkaloids, phenolics, terpenoids, and essential-oil constituents, that may act through complementary biological pathways [10,11]. Rather than serving merely as direct antibiotic substitutes, phytobiotics are increasingly regarded as components of integrated gut-health programs that may influence digestive secretion, epithelial renewal, microbial competitiveness, inflammatory tone, and resilience to dietary or environmental stressors [12,13]. Their practical value, however, depends on consistent efficacy under challenge conditions that closely resemble commercial production constraints.

Sanguinarine (SANG), a benzophenanthridine alkaloid predominantly derived from plants of the Papaveraceae family, particularly plume poppy (*Macleaya cordata)*, has emerged as a promising phytobiotic compound in poultry nutrition [14]. The biological relevance of SANG is associated with its capacity to interact with both microbial and host physiological processes, including intestinal development, microbial community structure, nutrient metabolism, and immune signaling [15]. In broilers, SANG-containing preparations have been associated with improved growth performance and enhanced intestinal immunity through modulation of mucosal inflammatory responses [15]. Moreover, SANG-related alkaloids, such as dihydrosanguinarine, have been reported to improve tryptophan metabolism and intestinal immune function through an aryl hydrocarbon receptor-mediated signaling pathway [16]. Evidence from inflammatory and necrotic enteritis models further suggests that plume poppy-derived additives may alleviate intestinal injury, modulate gut microbial ecology, and improve host metabolic responses under enteric stress [17,18]. These findings provide a biologically plausible basis for evaluating SANG as a targeted dietary intervention during *C. perfringens*-associated intestinal challenge.

Despite growing evidence supporting the beneficial effects of SANG, responses to phytobiotic additives can vary depending on inclusion level, bird age, health status, diet composition, and the severity of pathogen exposure [19]. Moreover, previous studies have shown that plume poppy-derived alkaloids can improve growth performance and intestinal health in broilers challenged with necrotic enteritis and modulate inflammatory and antioxidant responses under other enteric challenge conditions [20,21]. Nevertheless, the dose-dependent efficacy of SANG during *C. perfringens* challenge remains insufficiently defined, particularly when evaluated against a conventional antibiotic growth promoter within the same experimental model. Such comparative evaluation addresses an important gap by defining the inclusion level at which SANG most consistently supports productive, physiological, and intestinal outcomes under *C. perfringens* challenge.

Based on these considerations, it was hypothesized that dietary SANG supplementation would attenuate the adverse effects of *C. perfringens* challenge in broiler chickens by limiting pathogen-associated intestinal dysfunction, supporting mucosal development, improving redox and immune status, and consequently maintaining productive efficiency. Accordingly, the present study evaluated the effects of graded dietary SANG supplementation in broilers subjected to *C. perfringens* challenge. Its efficacy was compared with avilamycin, a conventional antibiotic growth promoter, through an integrated assessment of growth performance, carcass characteristics, biochemical indices, antioxidant capacity, immune status, and intestinal morphology and bacterial populations.

## 2. Materials and Methods

### 2.1. Bird Treatments and Management

A total of 360 newly hatched, mixed-sex Ross 308 broiler chicks, with an initial body weight of 42.9 ± 1.6 g, were selected for the experiment. Before allocation, all chicks were screened for the presence of *C. perfringens* using the cloacal swabbing technique [22], and only birds confirmed to be free of *C. perfringens* were included. The chicks were then randomly assigned to six experimental treatments in a completely randomized design. Each treatment included 10 replications, with six birds per replicate. The treatment groups were arranged as follows: a negative control group fed a basal diet without *C. perfringens* challenge; a positive control group fed a basal diet and challenged with *C. perfringens*; a challenged group fed a basal diet supplemented with 0.10 g/kg avilamycin (Maxus, Elanco Animal Health Ltd., Vienna, Austria); and three challenged groups fed basal diets supplemented with SANG (Sangrovit Extra; Phytobiotics Futterzusatzstoffe GmbH, Eltville, Germany) at 0.06, 0.09, or 0.12 g/kg diet.

The enteric challenge was induced as previously described [23], with minor modifications. Briefly, on day 7, all birds except those in the negative control group received an oral administration of *Eimeria* spp. at 10 times the manufacturer’s recommended dose of the commercial coccidiosis vaccine (COC-CIVAC-D2; Merck Animal Health, Madison, NJ, USA). Subsequently, on days 10, 11, and 12, birds in all challenged groups received *C. perfringens* type A (Microbiologics Inc., St. Cloud, MN, USA) by oral gavage at a concentration of 4 × 10^8^ CFU/mL. The establishment of the challenge was further supported by necropsy findings in birds that died following *C. perfringens* inoculation. Maxus was used as the reference antibiotic growth promoter. Its active compound, avilamycin, belongs to the orthosomycin class of antibiotics and is primarily active against susceptible Gram-positive bacteria, although susceptibility varies among bacterial taxa [24].

Sangrovit Extra is a standardized phytogenic feed additive containing an extract and processed leaves derived from plume poppy. The additive is formulated to contain 1.25% of the sum of four principal alkaloids (sanguinarine, chelerythrine, protopine, and allocryptopine), with SANG used as the principal marker compound at a target concentration of 0.5% [14]. Thus, based on the standardized 0.5% SANG concentration, supplementation at 0.06, 0.09, and 0.12 g/kg product provided approximately 0.30, 0.45, and 0.60 mg SANG/kg complete diet, respectively. These graded inclusion levels were selected to encompass low, intermediate, and higher supplementation levels within the range previously evaluated for Sangrovit Extra in broiler chickens (45–150 mg/kg complete feed) [14], thereby allowing assessment of the response to increasing dietary inclusion under *C. perfringens* challenge.

Throughout the 35-day experimental period, birds were reared in an environmentally controlled room using a battery cage system and had ad libitum access to mash feed and fresh drinking water. Standard husbandry procedures, along with recommended environmental conditions, were maintained in accordance with established broiler management guidelines [25]. Corn–soybean meal-based diets were formulated for the starter, grower, and finisher phases, corresponding to days 0–10, 11–25, and 26–35, respectively, with the strain nutrient recommendations used as a reference for diet formulation [26]. The ingredient composition and nutrient levels of the experimental diets are presented in Table 1.

Body weight and feed intake were recorded on a replicate-cage basis throughout the experimental period. These data were used to calculate daily feed intake (DFI), average daily gain (ADG), feed conversion ratio (FCR), and the European production efficiency factor (EPEF) for days 0–10, 11–25, and 26–35, as well as cumulatively for days 0–35. Mortality was recorded throughout the experiment. The FCR was corrected for mortality by accounting for the body weight of birds that died during each respective period, whereas ADG was not separately adjusted for mortality. The EPEF was calculated using the following equation: EPEF = [livability (%) × body weight (kg) × 100]/[age (days) × FCR] [27].

### 2.2. Sampling and Measurements

At the end of the experimental period, on day 35, one bird was randomly selected from each replicate for sample collection. The collected samples were used to evaluate serum biochemical, antioxidant, and immune-related parameters, carcass traits, ileal histomorphology, and ileal bacterial populations.

Approximately 5 mL of blood was assembled from the jugular vein of each selected bird. Serum was then sequestered by centrifugation at 3000× *g* for 10 min at 4 °C and reserved at −20 °C before analyses. Serum concentrations of total protein, albumin, glucose, aspartate aminotransferase (AST), alanine aminotransferase (ALT), total antioxidant capacity (T-AOC), total superoxide dismutase (T-SOD), and glutathione reductase were assessed utilizing commercial diagnostic kits (Randox Laboratories Ltd., Crumlin, UK). Globulin concentration was calculated as the difference between total protein and albumin concentrations. In addition, serum levels of immunoglobulin Y (IgY), immunoglobulin A (IgA), interleukin-1 (IL-1), interleukin-2 (IL-2), interleukin-6 (IL-6), and interferon gamma (IFN-γ) were quantified employing commercial assay kits (MyBioSource, San Diego, CA, USA). All analyses were performed in accordance with the manufacturers’ protocols.

For carcass trait assessment, the selected birds were weighed individually before euthanasia, defeathered, and eviscerated. Dressing percentage was calculated as carcass weight relative to live body weight [28]. The weights of carcass cuts, including breast muscles and leg quarters; visceral organs, including the liver, spleen, bursa of Fabricius, and empty gizzard; and abdominal fat pads were recorded. These weights were then expressed as percentages of live body weight.

For histomorphological evaluation, approximately 2 cm tissue segments were collected from the initial part of the proximal ileum, immediately distal to Meckel’s diverticulum, gently rinsed with phosphate-buffered saline (pH 7.4), and fixed in 10% neutral-buffered formalin. Following fixation, the tissue samples were processed through routine histological procedures, including dehydration, clearing, and paraffin embedding. Sections of 5 μm thickness were then prepared from each paraffin-embedded tissue block, mounted on glass slides, and stained with hematoxylin and eosin [29]. For morphometric analysis, ten properly oriented crypt–villus units were selected from each section. All histomorphometric measurements were performed by an evaluator blinded to the dietary treatment allocation. Villus length (VL), villus width (VW), and crypt depth (CD) were measured using ImageJ software (version 1.54p, National Institutes of Health, Bethesda, MD, USA). The villus length-to-crypt depth (VL/CD) ratio was subsequently calculated. Apparent villus surface area (AVSA) was determined using the following formula: AVSA = 2π × (VW/2) × VL [30].

For bacteriological assessment, 1 g of ileal digesta was collected aseptically and transferred into a sterile test tube. Each sample was homogenized in 9 mL of sterile 0.85% saline solution, after which the resulting suspension was subjected to tenfold serial dilution. From the appropriate dilutions, 100 µL aliquots were plated onto selective agar media (Sigma-Aldrich Co., Saint Louis, MO, USA) for the enumeration of presumptive target bacterial populations. *Lactobacillus* spp., *Bifidobacterium* spp., and *C. perfringens* were cultured on de Man, Rogosa, and Sharpe agar; bifidus selective medium agar; and tryptose sulfite cycloserine agar, respectively. Plates were incubated anaerobically using an anaerobic jar system (Thermo Fisher Scientific Inc., Basingstoke, Hampshire, UK) at 37 °C for 48 h for *Lactobacillus* spp., 72 h for *Bifidobacterium* spp., and 24 h for *C. perfringens*. Meanwhile, *Escherichia coli* was cultured on tryptone bile X-glucuronide agar and incubated aerobically at 44 °C for 24 h. Following incubation, colonies with typical morphology for each target group were counted using a colony counter (Interscience for Microbiology, Saint Nom la Bretêche, France). Appropriate serial dilutions were used to obtain countable plates and minimize colony crowding or merging. The bacterial populations were expressed as log_10_ colony-forming units per gram of ileal digesta (log_10_ CFU/g).

### 2.3. Statistical Analysis

For the analysis of performance variables, each replicate was treated as the experimental unit, whereas individual birds served as the experimental units for all other measurements. Prior to statistical analysis, the data were assessed for normality using the Shapiro–Wilk test and for homogeneity of variance using Levene’s test. Statistical analyses were conducted using one-way analysis of variance through the general linear model procedure in SAS software, version 9.4 (SAS Institute Inc., Cary, NC, USA). When significant treatment effects were detected, Tukey’s honest significant difference test was applied for pairwise comparisons among means. Statistical significance was declared at *p* ≤ 0.05. Results are presented as least-square means with their corresponding pooled standard error of the mean.

## 3. Results

### 3.1. Growth Performance

Growth performance results are presented in Table 2 and Table 3. During the starter phase, from days 0 to 10, dietary treatment had no significant effect on DFI, ADG, FCR, or EPEF (*p* > 0.05). During days 11 to 25, DFI remained unaffected by treatment (*p* > 0.05); however, ADG, FCR, and EPEF differed significantly among treatments (*p* ≤ 0.05). Birds in the positive control group showed lower ADG than those in the negative control group, whereas the avilamycin- and SANG-supplemented groups showed intermediate values that did not differ significantly from either control. The positive control group had the numerically highest FCR, while the negative control, avilamycin, and all SANG-supplemented groups showed comparable FCR values. The EPEF was lower in the positive control group than in the negative control, avilamycin, and 0.06 and 0.09 g/kg SANG groups. Birds receiving 0.12 g/kg SANG showed an intermediate EPEF response.

During the finisher phase, from days 26 to 35, no treatment effect was detected for DFI (*p* > 0.05). In contrast, ADG, FCR, and EPEF were significantly affected by treatment (*p* ≤ 0.05). Birds in the positive control group had lower ADG than those in the negative control group, whereas the avilamycin- and SANG-supplemented groups showed intermediate values. The FCR was higher in the positive control group than in the negative control, avilamycin, and 0.12 g/kg SANG groups, while the 0.06 and 0.09 g/kg SANG groups showed intermediate values. Similarly, EPEF was lower in the positive control group than in the negative control, avilamycin, and 0.06 and 0.12 g/kg SANG groups; birds receiving 0.09 g/kg SANG showed an intermediate EPEF value. Over the entire experimental period, from days 0 to 35, DFI did not differ among treatments (*p* > 0.05). In contrast, ADG, FCR, and EPEF were significantly affected by treatment (*p* = 0.001). The *C. perfringens*-challenged positive control group had lower ADG and EPEF and a higher FCR than all other groups. Supplementation with avilamycin or any level of SANG restored these variables to values comparable to those observed in the negative control group.

### 3.2. Carcass Characteristics

As shown in Table 4, dietary treatment affected dressing percentage, breast yield, and relative bursa weight (*p* ≤ 0.05), but had no significant effect on the relative weights of the leg, abdominal fat, liver, spleen, or gizzard (*p* > 0.05). The positive control group had a lower dressing percentage than the negative control, avilamycin, and 0.09 and 0.12 g/kg SANG groups. The 0.06 g/kg SANG group had an intermediate dressing percentage. Breast yield was lower in the positive control group than in all other groups, whereas avilamycin and all three SANG supplementation levels maintained breast yield at levels comparable with the negative control. Relative bursa weight was greater in birds supplemented with 0.06, 0.09, or 0.12 g/kg SANG than in the negative control, positive control, and avilamycin groups.

### 3.3. Biochemical Indicators and Antioxidant Status

Serum biochemical and antioxidant indices are presented in Table 5. Dietary treatment significantly affected serum total protein, globulin, glucose, AST, ALT, T-AOC, and T-SOD (*p* ≤ 0.05), whereas serum albumin and glutathione reductase activity were not affected by treatment (*p* > 0.05). The positive control group had lower serum total protein and glucose concentrations than all other groups. Supplementation with avilamycin or any level of SANG restored both variables to values comparable to those observed in the negative control group. Serum globulin concentration was lower in the positive control group than in the negative control and all SANG-supplemented groups, while the avilamycin group showed an intermediate value.

Serum AST activity was higher in the positive control group than in the negative control, avilamycin, and 0.09 and 0.12 g/kg SANG groups, whereas the 0.06 g/kg SANG group showed an intermediate value. The activity of ALT was higher in the positive control group than in the negative control group, while the avilamycin- and SANG-supplemented groups showed intermediate values. The positive control group had lower T-AOC than all other groups; however, avilamycin and all SANG supplementation levels restored T-AOC to values comparable to those of the negative control. The activity of T-SOD was also lower in the positive control group than in all other groups. The avilamycin group had greater T-SOD activity than the positive control group but lower activity than the negative control and all SANG-supplemented groups. In contrast, T-SOD activity in all SANG groups was comparable to that of the negative control group.

### 3.4. Intestinal Morphology

Dietary treatment significantly affected VL, CD, VL/CD ratio, VW, and AVSA (*p* ≤ 0.05; Table 6). Specifically, the positive control group had shorter villi than all other groups. Avilamycin supplementation restored VL to a value comparable with the negative control, whereas all SANG-supplemented groups had greater VL than both control groups and the avilamycin group. Similarly, CD was greater in the positive control group than in all other groups. Avilamycin reduced CD relative to the positive control, although its value remained greater than those of the negative control and SANG-supplemented groups. All SANG treatments restored CD to values comparable with the negative control.

The VL/CD ratio was also lower in the positive control group than in all other groups. Avilamycin increased this ratio relative to the positive control, but the value remained lower than those recorded in the negative control and SANG-supplemented groups. All SANG supplementation levels produced ratios comparable with the negative control. Furthermore, VW was lower in the positive control group than in all other groups, with no significant differences among the negative control, avilamycin, and SANG treatments. Regarding AVSA, values were greater in birds receiving 0.06, 0.09, or 0.12 g/kg SANG than in the positive control group, whereas those in the negative control and avilamycin groups were intermediate.

### 3.5. Immune Response

Serum immunoglobulin and cytokine concentrations differed significantly among treatments (*p* ≤ 0.01; Table 7). Specifically, the positive control group had a lower IgY concentration than all other groups. Conversely, supplementation with avilamycin or any level of SANG restored IgY to values comparable with the negative control. Similarly, IgA concentration was lower in the positive control group than in the negative control and 0.12 g/kg SANG groups. Meanwhile, the avilamycin group showed an intermediate IgA concentration that did not differ from the positive control, whereas the 0.06 and 0.09 g/kg SANG groups had values intermediate between the positive and negative controls. Regarding pro-inflammatory cytokines, the positive control group had the highest IL-1 concentration. Although avilamycin reduced IL-1 relative to the positive control, its concentration remained higher than those observed in the negative control and SANG-supplemented groups. By contrast, supplementation with 0.06 or 0.09 g/kg SANG restored IL-1 to values comparable with the negative control, while 0.12 g/kg SANG further reduced IL-1 below the level observed in all other groups.

For IL-2, concentrations were lower in the positive control and avilamycin groups than in the negative control and all SANG-supplemented groups. All SANG treatments maintained IL-2 concentrations comparable with the negative control. A similar pattern was observed for IL-6, for which the positive control group had the highest concentration, followed by the avilamycin group. Conversely, all SANG treatments reduced IL-6 relative to both the positive control and avilamycin groups. The 0.06 and 0.09 g/kg SANG groups showed IL-6 concentrations that did not differ significantly from the negative control, whereas the 0.12 g/kg SANG group remained higher than the negative control. Likewise, IFN-γ concentration was higher in the positive control and avilamycin groups than in the negative control and all SANG-supplemented groups. Nevertheless, all SANG supplementation levels restored IFN-γ to values comparable with those of the negative control.

### 3.6. Intestinal Bacterial Populations

Dietary treatment significantly affected all measured ileal bacterial populations (*p* = 0.001; Table 8). The positive control group had a greater *Escherichia coli* enumeration than all other groups. In contrast, avilamycin and all SANG supplementation levels reduced *Escherichia coli* enumerations relative to the positive control. Birds receiving 0.12 g/kg SANG had a lower enumeration than the negative control, while the 0.06 and 0.09 g/kg SANG groups had values comparable with both the negative control and avilamycin groups. Similarly, the positive control group had the highest *C. perfringens* enumeration, while the negative control had the lowest enumeration. Although avilamycin and all SANG treatments reduced *C. perfringens* enumerations relative to the positive control, their values remained higher than that of the negative control. Nevertheless, no significant differences were detected among the avilamycin and SANG-supplemented groups.

In contrast, the abundance of *Bifidobacterium* spp. was lower in the positive control group than in all other groups. Avilamycin increased the *Bifidobacterium* spp. population relative to the positive control, but its value remained lower than those observed in all SANG groups. Supplementation with 0.09 or 0.12 g/kg SANG produced *Bifidobacterium* spp. populations comparable with the negative control, while the 0.06 g/kg SANG group had an intermediate value. Likewise, the positive control and avilamycin groups had lower *Lactobacillus* spp. populations than the negative control and SANG-supplemented groups. The 0.09 g/kg SANG group had a greater *Lactobacillus* spp. population than the negative control, positive control, and avilamycin groups. Meanwhile, the 0.06 and 0.12 g/kg SANG groups showed intermediate populations that did not differ significantly from either the negative control or the 0.09 g/kg SANG group.

## 4. Discussion

The present study investigated the efficacy of graded dietary SANG supplementation in mitigating *C. perfringens*-induced enteric challenge in broiler chickens, with particular emphasis on productive performance, metabolic status, antioxidant capacity, immune response, and intestinal health. The results indicate that dietary SANG, particularly at inclusion rates of 0.09–0.12 g/kg, attenuated the adverse effects of *C. perfringens* challenge, with several outcomes comparable to or more favorable than those observed with the conventional antibiotic growth promoter avilamycin.

The impairment in growth performance following *C. perfringens* challenge is consistent with previous reports showing reduced nutrient utilization and growth efficiency during necrotic enteritis and subclinical infection [7,31]. Such performance losses have been associated with intestinal injury, diminished absorptive capacity, greater metabolic demands of the immune response, and competition for nutrients within the intestinal environment [32]. Dietary SANG supplementation improved cumulative ADG, FCR, and EPEF across all tested inclusion levels, producing responses comparable with both the negative control and avilamycin treatment. This agrees with earlier studies reporting growth-promoting effects of plume poppy-derived alkaloids in broilers under normal and challenged conditions [15,33]. The comparable performance response to SANG and avilamycin suggests that the benefits of SANG may involve broader effects on intestinal function, including nutrient utilization and modulation of the intestinal environment, as proposed previously [34].

Carcass responses further support the favorable effect of SANG on productive outcomes during *C. perfringens* challenge. The reductions in dressing percentage and breast yield in challenged birds are consistent with impaired tissue accretion under enteric stress, whereas SANG supplementation maintained breast yield and, at 0.09 and 0.12 g/kg, restored dressing percentage. These responses may be associated with improved protein utilization and reduced catabolic pressure during infection, as previously proposed [20]. The greater relative bursa weight observed in SANG-supplemented birds is also relevant because the bursa of Fabricius plays a central role in humoral immune development [35]. Together with the favorable changes in serum IgY and cytokine concentrations, this response is consistent with improved immune status during enteric challenge, although bursa weight is best interpreted as a supportive indicator rather than a direct measure of immune competence.

The serum biochemical changes observed following *C. perfringens* challenge indicate disruption of metabolic and hepatic homeostasis, consistent with previous reports of impaired protein metabolism and altered liver-associated indices during enteric infection in poultry [36,37]. Lower total protein and globulin concentrations may reflect altered nutrient utilization and increased metabolic demands associated with the host response to infection, whereas elevated AST and ALT activities are consistent with greater hepatic stress [38]. Dietary SANG supplementation improved these biochemical indices, with total protein and glucose returning to values comparable with the negative control and globulin showing a more consistent response than with avilamycin. The recovery of serum protein-related indices is consistent with improved metabolic stability and the previously reported physiological effects of SANG on intestinal and systemic health [39]. The restoration of globulin is particularly relevant because this fraction includes proteins involved in host defense, including immunoglobulins and acute-phase proteins [40], and therefore complements the favorable immune-related responses observed in SANG-supplemented birds. In addition, the normalization of AST activity, particularly at 0.09 and 0.12 g/kg SANG, is consistent with the antioxidant and anti-inflammatory properties previously attributed to SANG and related benzophenanthridine alkaloids [41].

Oxidative stress is a recognized component of enteric infection and is associated with increased reactive oxygen species production and depletion of endogenous antioxidant defenses [42]. The reduction in serum T-AOC and T-SOD following *C. perfringens* challenge therefore indicates impaired antioxidant status and agrees with previous reports in broilers affected by necrotic enteritis [43,44]. Dietary SANG supplementation improved both indices, with T-SOD restored to values comparable with the negative control and exceeding those observed with avilamycin. This response is consistent with the antioxidant properties previously attributed to SANG and related benzophenanthridine alkaloids, including free-radical scavenging activity and enhancement of endogenous antioxidant defenses [45]. Improved redox status may also contribute to the preservation of intestinal and systemic tissue function during enteric challenge, given the established role of oxidative injury in *C. perfringens*-associated intestinal pathology [46]. Collectively, these findings support antioxidant modulation as a plausible contributor to the beneficial effects of SANG under enteric challenge conditions.

The intestinal epithelium is central to nutrient absorption and barrier function, and disruption of its architecture is a characteristic consequence of *C. perfringens* challenge [47]. The reductions in VL, VL/CD ratio, VW, and AVSA, together with increased CD, are consistent with epithelial injury and altered mucosal renewal reported during enteric infection [7]. Dietary SANG supplementation produced a marked improvement in ileal architecture, particularly through greater villus development and normalization of crypt depth and the VL/CD ratio. These changes are compatible with improved absorptive capacity and may partly explain the parallel recovery in growth performance. The lower crypt depth observed in SANG-supplemented birds may also reflect reduced demand for compensatory epithelial renewal under conditions of diminished mucosal injury [48]. The favorable histomorphological response is consistent with previous studies linking plume poppy-derived alkaloids with epithelial migration, mucosal repair, and improved barrier-related function [17,49,50]. Collectively, these findings suggest that the intestinal effects of SANG extend beyond suppression of harmful bacterial populations and may contribute to greater mucosal stability and resilience during enteric challenge.

The immune response to *C. perfringens* challenge is characterized by coordinated innate and adaptive responses, with excessive inflammation contributing to intestinal injury and impaired performance [51]. The elevated IL-1, IL-6, and IFN-γ concentrations together with reduced IgY and IgA in challenged birds are consistent with an inflammatory response accompanied by impaired humoral immune status [52]. Dietary SANG supplementation improved this immune profile by restoring IgY and, at the highest inclusion level, IgA, while reducing the concentrations of pro-inflammatory cytokines. These responses are consistent with the immunomodulatory and anti-inflammatory properties previously attributed to SANG and related alkaloids, including effects on NF-κB- and MAPK-associated signaling pathways [16,53]. A lower pro-inflammatory cytokine burden may favor preservation of epithelial integrity, whereas improved immunoglobulin status may support host defense against *C. perfringens* and its toxins [54,55]. Overall, the coordinated changes in cytokines and immunoglobulins indicate that SANG was associated with a more balanced immune response during enteric challenge, in agreement with the broader immunomodulatory effects described for phytobiotic compounds in poultry [11].

Changes in ileal bacterial populations indicate that modulation of the intestinal microbial environment may contribute to the beneficial responses associated with SANG supplementation. The increase in *C. perfringens* and *Escherichia coli* populations and the concurrent reduction in beneficial bacteria in challenged birds are consistent with enteric dysbiosis, which has been associated with epithelial injury, inflammation, and impaired nutrient utilization [56]. Both avilamycin and SANG reduced harmful bacterial populations, whereas SANG showed a more favorable effect on beneficial bacteria, particularly *Bifidobacterium* spp. and *Lactobacillus* spp. These findings agree with previous reports describing microbiota-modulating effects of SANG in broilers [15,57]. The greater abundance of these beneficial genera may support intestinal homeostasis through functions associated with barrier maintenance, inflammatory regulation, and nutrient utilization [58]. Their production of short-chain fatty acids, bacteriocins, and other antimicrobial metabolites may also contribute to competitive interactions that limit *C. perfringens* persistence within the intestinal environment [18].

Several limitations of the present study should be considered when interpreting the intestinal responses. Although the *C. perfringens* challenge produced clear alterations in productive performance, systemic inflammatory and antioxidant responses, ileal morphology, and bacterial enumeration, disease severity was not directly characterized using standardized intestinal lesion scoring, toxin quantification, or molecular markers of epithelial injury. Consequently, the observed histomorphological and physiological responses should be interpreted as evidence of enteric disturbance rather than as direct quantitative measures of disease severity. Future studies should incorporate lesion scoring, toxin measurements, and molecular assessments of intestinal barrier integrity and tissue injury to strengthen characterization of the challenge model and the protective effects of SANG. In addition, the microbiological assessment was restricted to culture-based enumeration of selected bacterial groups. Although this approach provides useful quantitative information on targeted, biologically relevant populations, it does not capture the full complexity of the intestinal microbiota and should not be considered a comprehensive assessment of microbial community structure. High-throughput approaches, including 16S rRNA gene sequencing or shotgun metagenomic analysis, would provide a more comprehensive assessment of microbial diversity, community composition, and functional changes and could further clarify the relationship between SANG supplementation, intestinal microbial ecology, and host responses during *C. perfringens* challenge. A further limitation concerns the anatomical location used for histomorphological evaluation. Tissue samples were collected only from the initial portion of the proximal ileum; therefore, the morphological measurements obtained in this study should be interpreted as region-specific and should not be considered representative of the entire ileum. Because intestinal morphology may vary along the length of the ileum, future studies should include additional ileal regions, particularly the mid-ileum, to provide a more comprehensive assessment of regional morphological responses.

## 5. Conclusions

Overall, dietary SANG supplementation mitigated the adverse effects of *C. perfringens*-induced enteric challenge in broiler chickens, with favorable responses in growth performance, metabolic status, antioxidant capacity, immune response, intestinal morphology, and ileal bacterial populations. Among the tested inclusion levels, 0.09 and 0.12 g/kg generally produced the most consistent responses, with several outcomes comparable with or superior to those observed with avilamycin. The concurrent improvements in bacterial balance, inflammatory and antioxidant status, and intestinal architecture are consistent with the antimicrobial, immunomodulatory, antioxidant, and gut-supportive properties previously attributed to SANG. These findings support its potential as a non-antibiotic nutritional strategy for improving broiler resilience under enteric challenge conditions. Further research under commercial production settings is warranted to confirm its practical efficacy and to clarify the longer-term relationships among SANG supplementation, intestinal microbial ecology, metabolic responses, and immune development.

## Figures and Tables

**Table 1:** Ingredient and nutritional composition of the experimental diets (as-fed basis).

Ingredient (%)	Starter (0–10 d)	Grower (11–25 d)	Finisher (26–35 d)
Yellow corn	51.58	58.5	59.82
Soybean meal	32.4	28.2	27.0
Corn oil	3.3	3.6	4.34
Corn gluten meal	6.3	4.71	5.1
Wheat bran	2.0	1.0	0.0
Dicalcium phosphate	2.05	1.82	1.68
Ground limestone	0.9	0.88	0.87
Choline chloride	0.05	0	0
DL-methionine	0.3	0.26	0.25
L-lysine	0.38	0.3	0.26
Salt	0.4	0.4	0.4
Threonine	0.14	0.13	0.08
Vitamin–mineral premix	0.2	0.2	0.2
Calculated nutrient composition			
Metabolizable energy, kcal/kg	3000	3100	3200
Crude protein, %	23.0	21.5	20.0
Non-phytate phosphorus, %	0.48	0.435	0.405
Calcium, %	0.96	0.87	0.81
Digestible lysine, %	1.28	1.15	1.06
Digestible sulfur amino acids, %	0.95	0.85	0.83
Digestible threonine, %	0.86	0.77	0.71
Sodium, %	0.17	0.17	0.17
Potassium, %	0.87	0.79	0.76
Chloride, %	0.26	0.25	0.25
Dietary electrolyte balance, mEq/kg	223	205	198

The vitamin–mineral premix supplied the following quantities per kilogram of premix: vitamin A, 12,000,000 IU; vitamin D_3_, 5,000,000 IU; vitamin E, 80,000 IU; vitamin K_3_, 3200 mg; vitamin B_1_, 3200 mg; vitamin B_2_, 8600 mg; vitamin B_3_, 65,000 mg; pantothenic acid, 20,000 mg; vitamin B_6_, 4300 mg; biotin, 220 mg; vitamin B_9_, 2200 mg; vitamin B_12_, 17 mg; antioxidant (butylated hydroxyanisole and butylated hydroxytoluene), 50,000 mg; copper, 16,000 mg; iodine, 1250 mg; iron, 20,000 mg; manganese, 120,000 mg; selenium, 300 mg; and zinc, 110,000 mg. Dietary electrolyte balance was calculated as sodium + potassium − chloride on a milliequivalent basis.

**Table 2:** Effects of dietary sanguinarine (SANG) supplementation on growth performance of broiler chickens challenged with *Clostridium perfringens* (*C. perfringens*) during the starter and grower phases.

Period/ Variable	NC	PC	AVM	SANG06	SANG09	SANG12	SEM	*p*-Value
0–10 d								
DFI (g)	19.5	19.8	19.9	19.9	19.9	19.5	0.39	NS
ADG (g)	18.2	17.8	18.2	18.0	18.0	18.1	0.55	NS
FCR (g:g)	1.079	1.117	1.096	1.109	1.106	1.085	0.019	NS
EPEF (%)	235.1	217.6	230.9	224.6	224.9	234.1	10.69	NS
11–25 d								
DFI (g)	90.8	89.9	90.2	90.3	90.6	90.0	1.27	NS
ADG (g)	75.7 ^a^	68.6 ^b^	74.4 ^ab^	74.1 ^ab^	74.9 ^ab^	73.2 ^ab^	1.45	0.05
FCR (g:g)	1.200 ^b^	1.320 ^ab^	1.214 ^b^	1.218 ^b^	1.211 ^b^	1.231 ^b^	0.017	0.001
EPEF (%)	448.8 ^a^	388.4 ^b^	444.4 ^a^	439.9 ^a^	446.4 ^a^	431.8 ^ab^	11.54	0.01

Data were analyzed by one-way ANOVA followed by Tukey’s HSD test. Within a row, means with different superscript letters differ at *p* ≤ 0.05. SEM, pooled standard error of the mean (*n* = 10 replicates/treatment); NS, not significant. NC, non-challenged negative control; PC, *C. perfringens*-challenged positive control; AVM, PC + 0.10 g/kg avilamycin; SANG06, SANG09, and SANG12, PC + 0.06, 0.09, and 0.12 g/kg SANG, respectively. DFI, daily feed intake; ADG, average daily gain; FCR, feed conversion ratio; EPEF, European production efficiency factor.

**Table 3:** Effects of dietary sanguinarine (SANG) supplementation on growth performance of broiler chickens challenged with *Clostridium perfringens* (*C. perfringens*) during the finisher and cumulative phases.

Period/ Variable	NC	PC	AVM	SANG06	SANG09	SANG12	SEM	*p*-Value
26–35 d								
DFI (g)	150.4	148.9	148.5	149.1	150.5	149.9	2.11	NS
ADG (g)	102.3 ^a^	93.9 ^b^	99.8 ^ab^	99.1 ^ab^	98.5 ^ab^	100.6 ^ab^	1.64	0.05
FCR (g:g)	1.473 ^b^	1.589 ^a^	1.489 ^b^	1.506 ^ab^	1.528 ^ab^	1.491 ^b^	0.04	0.05
EPEF (%)	453.6 ^a^	407.7 ^b^	453.3 ^a^	444.3 ^a^	437.4 ^ab^	443.0 ^a^	7.53	0.001
0–35 d								
DFI (g)	87.5	86.7	86.8	86.9	87.5	87.0	1.00	NS
ADG (g)	66.9 ^a^	61.3 ^b^	65.6 ^a^	65.2 ^a^	65.4 ^a^	65.3 ^a^	0.79	0.001
FCR (g:g)	1.308 ^b^	1.416 ^a^	1.323 ^b^	1.334 ^b^	1.339 ^b^	1.332 ^b^	0.012	0.001
EPEF (%)	509.7 ^a^	457.2 ^b^	510.1 ^a^	501.6 ^a^	498.8 ^a^	495.8 ^a^	8.27	0.001

Data were analyzed by one-way ANOVA followed by Tukey’s HSD test. Within a row, means with different superscript letters differ at *p* ≤ 0.05. SEM, pooled standard error of the mean (*n* = 10 replicates/treatment); NS, not significant. NC, non-challenged negative control; PC, *C. perfringens*-challenged positive control; AVM, PC + 0.10 g/kg avilamycin; SANG06, SANG09, and SANG12, PC + 0.06, 0.09, and 0.12 g/kg SANG, respectively. DFI, daily feed intake; ADG, average daily gain; FCR, feed conversion ratio; EPEF, European production efficiency factor.

**Table 4:** Effects of dietary sanguinarine (SANG) supplementation on relative weight (% of live body weight (BW)) of carcass yields and visceral organs in broiler chickens challenged with *Clostridium perfringens* (*C. perfringens*) at 35 days of age.

Variable, % Live BW	NC	PC	AVM	SANG06	SANG09	SANG12	SEM	*p*-Value
Dressing percentage	71.2 ^a^	69.2 ^b^	71.2 ^a^	70.3 ^ab^	70.8 ^a^	70.8 ^a^	0.29	0.001
Breast	27.8 ^a^	24.7 ^b^	27.1 ^a^	26.9 ^a^	27.0 ^a^	26.9 ^a^	0.57	0.007
Leg	20.4	20.1	20.7	20.1	20.4	20.6	0.33	NS
Abdominal fat	0.95	0.79	0.83	0.99	0.81	0.99	0.11	NS
Liver	2.01	2.01	2.02	2.03	2.02	2.00	0.07	NS
Bursa	0.16 ^b^	0.18 ^b^	0.19 ^b^	0.24 ^a^	0.23 ^a^	0.24 ^a^	0.002	0.05
Spleen	0.10	0.11	0.13	0.12	0.11	0.09	0.01	NS
Gizzard	1.74	2.09	2.20	2.37	2.13	2.13	0.15	NS

Data were analyzed by one-way ANOVA followed by Tukey’s HSD test. Within a row, means with different superscript letters differ at *p* ≤ 0.05. SEM, pooled standard error of the mean (*n* = 10 birds/treatment); NS, not significant. NC, non-challenged negative control; PC, *C. perfringens*-challenged positive control; AVM, PC + 0.10 g/kg avilamycin; SANG06, SANG09, and SANG12, PC + 0.06, 0.09, and 0.12 g/kg SANG, respectively.

**Table 5:** Effects of dietary sanguinarine (SANG) supplementation on serum biochemistry and antioxidant capacity in broiler chickens challenged with *Clostridium perfringens* (*C. perfringens*) at 35 days of age.

Variable	NC	PC	AVM	SANG06	SANG09	SANG12	SEM	*p*-Value
Biochemical indices								
TP (g/dL)	5.90 ^a^	4.99 ^b^	5.74 ^a^	5.87 ^a^	5.79 ^a^	5.81 ^a^	0.16	0.001
ALB (g/dL)	2.88	2.91	2.89	2.69	2.72	2.76	0.06	NS
GLO (g/dL)	3.02 ^a^	2.08 ^b^	2.85 ^ab^	3.18 ^a^	3.07 ^a^	3.05 ^a^	0.26	0.05
GLU (mg/dL)	209 ^a^	177 ^b^	207 ^a^	208 ^a^	210 ^a^	208 ^a^	4.73	0.001
Liver function								
AST (U/L)	47.6 ^b^	55.8 ^a^	48.8 ^b^	51.1 ^ab^	49.3 ^b^	48.5 ^b^	1.63	0.01
ALT (U/L)	8.04 ^b^	10.72 ^a^	8.39 ^ab^	8.61 ^ab^	8.37 ^ab^	9.26 ^ab^	0.53	0.01
Antioxidant capacity								
T-AOC (U/mL)	2.35 ^a^	1.88 ^b^	2.18 ^a^	2.31 ^a^	2.24 ^a^	2.16 ^a^	0.09	0.01
T-SOD (U/mL)	188 ^a^	168 ^c^	175 ^b^	192 ^a^	187 ^a^	183 ^a^	3.24	0.01
GR (U/mL)	3.45	3.31	3.40	2.77	3.08	2.28	0.29	NS

Data were analyzed by one-way ANOVA followed by Tukey’s HSD test. Within a row, means with different superscript letters differ at *p* ≤ 0.05. SEM, pooled standard error of the mean (*n* = 10 birds/treatment); NS, not significant. NC, non-challenged negative control; PC, *C. perfringens*-challenged positive control; AVM, PC + 0.10 g/kg avilamycin; SANG06, SANG09, and SANG12, PC + 0.06, 0.09, and 0.12 g/kg SANG, respectively. TP, total protein; ALB, albumin; GLO, globulin; GLU, glucose; AST, aspartate aminotransferase; ALT, alanine aminotransferase; T-AOC, total antioxidant capacity; T-SOD, total superoxide dismutase; GR, glutathione reductase.

**Table 6:** Effects of dietary sanguinarine (SANG) supplementation on ileal histomorphology in broiler chickens challenged with *Clostridium perfringens* (*C. perfringens*) at 35 days of age.

Variable	NC	PC	AVM	SANG06	SANG09	SANG12	SEM	*p*-Value
VL (µm)	680 ^b^	518 ^c^	665 ^b^	712 ^a^	723 ^a^	709 ^a^	19.21	0.001
CD (µm)	132 ^c^	156 ^a^	143 ^b^	130 ^c^	129 ^c^	131 ^c^	3.31	0.01
VL/CD	5.15 ^a^	3.32 ^c^	4.65 ^b^	5.48 ^a^	5.60 ^a^	5.41 ^a^	0.33	0.05
VW (µm)	70.5 ^a^	59.8 ^b^	69.2 ^a^	71.1 ^a^	72.2 ^a^	72.0 ^a^	3.44	0.01
AVSA (mm^2^)	0.151 ^ab^	0.097 ^b^	0.145 ^ab^	0.159 ^a^	0.164 ^a^	0.160 ^a^	0.016	0.05

Data were analyzed by one-way ANOVA followed by Tukey’s HSD test. Within a row, means with different superscript letters differ at *p* ≤ 0.05. SEM, pooled standard error of the mean (*n* = 10 birds/treatment). NC, non-challenged negative control; PC, *C. perfringens*-challenged positive control; AVM, PC + 0.10 g/kg avilamycin; SANG06, SANG09, and SANG12, PC + 0.06, 0.09, and 0.12 g/kg SANG, respectively. VL, villus length; CD, crypt depth; VL/CD, villus length-to-crypt depth ratio; VW, villus width; AVSA, apparent villus surface area.

**Table 7:** Effects of dietary sanguinarine (SANG) supplementation on serum immunoglobulin and cytokine concentrations in broiler chickens challenged with *Clostridium perfringens* (*C. perfringens*) at 35 days of age.

Variable	NC	PC	AVM	SANG06	SANG09	SANG12	SEM	*p*-Value
Immunoglobulins								
IgY (µg/mL)	12.2 ^a^	9.2 ^b^	11.3 ^a^	11.4 ^a^	11.4 ^a^	11.7 ^a^	0.49	0.01
IgA (µg/mL)	47.8 ^a^	36.4 ^c^	40.3 ^bc^	45.2 ^ab^	43.4 ^ab^	46.2 ^a^	1.82	0.001
Cytokines								
IL-1 (pg/mL)	3158 ^c^	3973 ^a^	3566 ^b^	3168 ^c^	3237 ^c^	2868 ^d^	86.5	0.001
IL-2 (pg/mL)	9034 ^a^	7198 ^b^	7673 ^b^	8945 ^a^	8670 ^a^	8716 ^a^	171.1	0.001
IL-6 (pg/mL)	6688 ^d^	8551 ^a^	7846 ^b^	7146 ^cd^	6796 ^cd^	7297 ^c^	185.7	0.001
IFN-γ (pg/mL)	4080 ^b^	5477 ^a^	5298 ^a^	4250 ^b^	4349 ^b^	4192 ^b^	162.4	0.001

Data were analyzed by one-way ANOVA followed by Tukey’s HSD test. Within a row, means with different superscript letters differ at *p* ≤ 0.05. SEM, pooled standard error of the mean (*n* = 10 birds/treatment). NC, non-challenged negative control; PC, *C. perfringens*-challenged positive control; AVM, PC + 0.10 g/kg avilamycin; SANG06, SANG09, and SANG12, PC + 0.06, 0.09, and 0.12 g/kg SANG, respectively. IgY, immunoglobulin Y; IgA, immunoglobulin A; IL-1, interleukin-1; IL-2, interleukin-2; IL-6, interleukin-6; IFN-γ, interferon gamma.

**Table 8:** Effects of dietary sanguinarine (SANG) supplementation on ileal bacterial populations (log_10_ CFU/g) in broiler chickens challenged with *Clostridium perfringens* (*C. perfringens*) at 35 days of age.

Variable, log_10_ CFU/g	NC	PC	AVM	SANG06	SANG09	SANG12	SEM	*p*-Value
*Escherichia coli*	5.75 ^b^	6.61 ^a^	5.55 ^cd^	5.71 ^bc^	5.62 ^bcd^	5.53 ^d^	0.055	0.001
*C. perfringens*	1.64 ^c^	6.25 ^a^	2.85 ^b^	2.87 ^b^	2.61 ^b^	2.49 ^b^	0.146	0.001
*Bifidobacterium* spp.	8.6 ^a^	6.12 ^d^	6.86 ^c^	7.82 ^b^	7.99 ^ab^	8.37 ^ab^	0.223	0.001
*Lactobacillus* spp.	6.94 ^b^	6.19 ^c^	6.27 ^c^	6.95 ^ab^	7.13 ^a^	7.02 ^ab^	0.066	0.001

Data were analyzed by one-way ANOVA followed by Tukey’s HSD test. Within a row, means with different superscript letters differ at *p* ≤ 0.05. SEM, pooled standard error of the mean (*n* = 10 birds/treatment). NC, non-challenged negative control; PC, *C. perfringens*-challenged positive control; AVM, PC + 0.10 g/kg avilamycin; SANG06, SANG09, and SANG12, PC + 0.06, 0.09, and 0.12 g/kg SANG, respectively.

## Data Availability

The data are available upon reasonable request from the corresponding authors.
